# Red Cross Chairman's Proposal

**Published:** 1920-01-17

**Authors:** 


### MORE MONEY NEEDED BY HOSPITALS.

### Red Cross Chairman's Proposal.

THE following appeared in the *Times* of January 12, under the above heading :—

The hospitals of the country are faced with a serious financial situation. This state of things is due in the first place to the fact that the prices of everything used in these institutions have gone up so enormously that even those which before the war had sufficient revenue from endowments to meet expenditure are no longer in so favourable a position, and are compelled to appeal to the public for assistance. Another reason is that all the arrears of the past five years in the way of renewals, structural alterations—indeed, everything that a hospital requires—have now to be made good. It is for these reasons that so many hospitals are asking for large sums to meet capital expenditure. Among the London hospitals which are making appeals to the public are St. Bartholomew's and Middlesex; and St. Thomas's and St. George's will want money very soon, to say nothing of almost every country hospital.

In making this statement to a representative of the *Times* yesterday, Sir Arthur Stanley, Chairman of the British Red Cross Society, said it had occurred to him and some of his colleagues that a voluntary organisation such as the Joint Council of the British Red Cross Society and the Order of St. John—which is the peace-time successor of the Joint War Committee, and which, through the agency of the *Times*, raised so large a sum of money during the war for the sick and wounded of His Majesty's Forces—might be utilised to help the voluntary hospitals of the country. The London hospitals have King Edward's Fund, and other funds, such as the Hospital Sunday Fund, the Hospital Saturday Fund, and the League of Mercy; but no effort, so far, has been made to obtain money for country hospitals on any centralised basis.

"There are other ways," Sir Arthur Stanley explained, "in which hospitals can be helped. The value of comparative tables, whereby any hospital can see at a glance whether it is spending more or less in any particular branch of its work than the average spent by other hospitals, has been demonstrated both by King Edward's Fund, by the annual report published by the Joint War Committee of the British Red Cross and the Order of St. John, and by the tables of expenditure of over 1,600 auxiliary military hospitals. Moreover, it is hoped that it may be possible to establish central stores, such as the British Red Cross had during the war, to which contributions of garments, etc., can be made, and on which the voluntary hospitals can draw when needed. Another way in which the Red Cross can undoubtedly help voluntary hospitals is in stimulating local interest in any particular hospital.

"It is a well-known fact," added Sir Arthur, "that hospitals receive support from only about 10 per cent. of the population. If even only 20 per cent. more can be induced to take an interest in the hospitals of the country, that will go very far to meet the financial difficulties in which they find themselves at present, and will help to avert the danger of hospitals being taken over by the State, which is a thing that nobody desires to see, and least of all the State itself."

A meeting of the British Hospitals Association, over which Lord Sandhurst will preside, will be held on Friday, January 23, at 3 P.M., either at St. Bartholomew's or St. Thomas's Hospital, to consider the financial position of voluntary hospitals. At this meeting, at which representatives of all hospitals will attend, Sir Arthur Stanley will submit his proposals, and suggest that the Red Cross is one source from which help may be forthcoming. It is probable that "Our Day" may be repeated in October of this year in order to obtain money to carry on general Red Cross work, of which hospital work will then be a part. The Red Cross Society has already given to hospitals throughout the country £1,350,000 and £250,000 to the hospitals in London, out of the surplus funds raised during the war, and is now preparing the way for rendering still further assistance.

